# Broadening the Scope (Part II)…

**DOI:** 10.1096/fba.2020-00060

**Published:** 2020-09-06

**Authors:** Ralph A. Bradshaw, Philip D. Stahl

**Affiliations:** ^1^ University of California Irvine CA USA; ^2^ Washington University St. Louis MO USA

A few months ago, we announced that FASEB BioAdvances would be extending its coverage beyond just articles in biomedicine and biology to include reports on history, science policy and society and biomedical education. Since that time, we have had contributions in all three areas. We also indicated that we would be expanding this coverage, as well as for select areas of science that are rapidly developing, by the publication of focused sets of papers as special issues of the journal. These are envisioned to be ensembles of articles on a single theme that have been invited by one or more guest editors who will play the lead role in planning, inviting and assisting with the peer review process. Each topic will be approved and developed with the participation of the journal leadership. The size and scope of the initial solicitation is expected to be 10 and 15 articles of any type that we publish.

There will be two formats for how these focused groups of papers will appear in the journal: **special issues** and **special collections**. In the former case, accepted articles will appear in the preview lists (Accepted Papers and Early View) but will not appear in the regular pages of the journal until all the articles have been received and reviewed, at which time they will be published as one group in one of the monthly issues of the journal that will be devoted only to this set of papers. The copyedited papers that are found in the Early View list will contain an indication that the article will appear in due course in a special issue. With special collections, articles will also appear in the preview lists but then will be published in regular issues of the journal (under the appropriate T of C category) as they are received and accepted. Each such article will be 'tagged' (hyper‐linked) to indicate that it is part of a special topic project and will simultaneously also be collected at a separate site (linked from the regular pages of the journal) and this will be accessible from the point that the first article appears and will grow with the acceptance of each additional contribution. These will be designated as SPECIAL COLLECTIONS. This category, as opposed to the special issue option, has a number of attractive features. Collections will be more dynamic, flexible and can, with the consent of the guest editors, be extended beyond the original planned content if appropriate or desirable. It also makes the special collection more immediately available to readers (and hopefully will have the positive effect of encouraging stragglers to finish their contributions and get them in). FBA will keep both formats available as, depending on several variables, one might suit an individual project better than the other.

The first special collection on Biomedical Education, put together by guest editors Michael Hortsch, Jasna Markovac and James Woolliscroft, will become available with this issue of the journal, initiated by the article on STEM Crisis Teaching by Van Nuland, Hall and Langley. Over the next several months we expect this collection to increase to a dozen or more articles. At the same time, we are planning a special collection on exosomes and homeostasis, the influence of the nervous system on cancer and other pathologies and an additional education effort on managing medical curricula in the time of a pandemic. Working with the World NCD (Noncommunicable Disease) Federation (https://worldncdfederation.org/), we have also agreed to do a special issue of articles that represent many of the key lectures/panels of their canceled 2020 Congress, which we expect to appear in the journal by the end of the year.

Starting with this issue, we will also begin to display new monthly artwork for the digital cover of the journal that appears on the website. We will select each month’s cover from both new and archived material and we will welcome suggestions from authors from their submissions.

While we all are wrestling with the restrictions and disruptions wrought by COVID 19, we need to remember that it has never been more important to support the scientific research enterprise in all its manifestations because in the end, it is these efforts that will eventually control the pandemic and bring all of our lives back to normalcy.

We hope you all will remain healthy and productive in these challenging times.

